# Heat Shock Protein 90 and Role of Its Chemical Inhibitors in Treatment of Hematologic Malignancies

**DOI:** 10.3390/ph5080779

**Published:** 2012-07-25

**Authors:** Ngoc Ho, Adam Li, Shaoguang Li, Haojian Zhang

**Affiliations:** Division of Hematolog and Oncology, Department of Medicine, University of Massachusetts Medical School, 364 Plantation Street, Worcester, MA 01605, USA

**Keywords:** heat shock protein 90, chemical inhibitors, hematologic malignancies

## Abstract

Heat shock protein 90 (Hsp90) is a conserved and constitutively expressed molecular chaperone and it has been shown to stabilize oncoproteins and facilitate cancer development. Hsp90 has been considered as a therapeutic target for cancers and three classes of Hsp90 inhibitors have been developed: (1) benzoquinone ansamycin and its derivatives, (2) radicicol and its derivates, and (3) small synthetic inhibitors. The roles of these inhibitors in cancer treatment have been studied in laboratories and clinical trials, and some encouraging results have been obtained. Interestingly, targeting of Hsp90 has been shown to be effective in inhibition of cancer stem cells responsible for leukemia initiation and progression, providing a strategy for finding a cure. Because cancer stem cells are well defined in some human leukemias, we will focus on hematologic malignancies in this review.

## 1. Introduction

Heat Shock Protein 90 (Hsp90) is a highly conserved, constitutively expressed molecular chaperone that facilitates folding of its client proteins that are involved in signal transduction, protein trafficking, receptor maturation and innate and adaptive immunity [1]. As an ATPase, Hsp90 uses the energy generated in a complex cycle of adenosine triphosphate (ATP) binding and hydrolysis to assist protein folding. In these processes, client proteins recognition by Hsp90 is guided by co-chaperones, such as cell division cycle 37 (CDC37) and cyclin-dependent kinase inhibitor p23 [2]. It has been shown that cancer cells use the Hsp90 chaperone machinery to facilitate the function of numerous oncoproteins; therefore, Hsp90 is considered as an important therapeutic target in cancers, which has led to the development of Hsp90 inhibitors. Hsp90 inhibitors are a diverse group of novel agents that have been demonstrated to have pro-apoptotic effects on malignant cells through inhibition of ATP binding in the ATP/ADP-binding pocket of the heat shock protein. A considerable amount of research has been carried out to understand the role of Hsp90 in hematological malignancies [3,4,5,6]. Progress in the preclinical and clinical evaluation of Hsp90 inhibitors in hematological malignancies has also demonstrated the important role of Hsp90 in cancer development. This review describes recent advances in our understanding of Hsp90 biology and its inhibitors for the treatment of cancers, especially hematological malignancies and leukemia stem cells (LSCs).

## 2. Structure and Functional Regulation of Hsp90

### 2.1. Structure of Hsp90

The crystal structure of Hsp90 was first reported about 16 years ago [7]. However, its structural analysis has been hampered for a long time by the high intrinsic flexibility of structural conformation until recent studies uncovered the full-length structure of Hsp90 [8,9,10]. Hsp90 is a flexible dimer where the monomer has a highly conserved amino-terminal domain (NTD), a middle-domain and a carboxy-terminal domain (CTD). ATP binds to the NTD at the ATP-binding site, which consists of an α and β-sandwich motif in a deep ATP binding pocket. Upon ATP binding, an ATP lid composing of several-conserved amino acid residues in the NTD changes its position, resulting in the conformation change of the entire Hsp90 into a compact and twisted Hsp90 dimer known as the closed conformation. ATP hydrolysis is required for sustaining Hsp90’s essential function as it drives conformational changes in the Hsp90 chaperone cycle and restores Hsp90 to its open conformation [11]. A recent study has indicated that a charged linker that connects between the NTD and middle domain is an important regulator of Hsp90 function [12]. Deletion of this linker does not affect the structural integrity and ATP binding ability of Hsp90, but significantly reduces the ability of ATP hydrolysis due to the decreased sensitivity for Aha1 (Hsp90 ATPase homologue 1)-mediated ATPase acceleration. Therefore, deletion of this charged linker eventually causes a loss of Hsp90 regulation by co-chaperones. The middle domain consists of two αβα motifs that play an important role in client recognition. Last but not the least, CTD mediates dimerization of Hsp90 [13], as truncation of this region by digestion with the Ca^2+^-dependent protease m-calpain results in dissociation of the dimer.

### 2.2. Functional Regulation of Hsp90

Besides the conformational changes induced by ATP hydrolysis, the function of Hsp90 is also regulated by co-chaperones and post-translational modifications. Many co-chaperones that display multifaceted roles on Hsp90 function have been identified (Table 1). Some known co-chaperones, such as Cdc37, p23, ubiquitin ligase-associated protein SGT1, and Aha1, affect the ATPase activity of Hsp90 by regulating its conformational dynamics through several different mechanisms. Aha1 acts as an activator and enhances Hsp90’s ATPase by stimulating the early conformational transitions of the ATPase cycle [14]. Cdc37, p23 and SGT1 inhibit the ATPase activity of Hsp90 through multiple mechanisms. For example, Cdc37 can block the ATP-binding pocket and preclude NTD dimerization [15,16], whereas p23 can reduce the conformational flexibility by binding to the Hsp90 NTD [9]. Another class of co-chaperones functions as an adaptor and delivers substrates to Hsp90. Cdc37 links protein kinases to Hsp90 [17], and homeobox HOP delivers progesterone hormone receptor to Hsp90 [18]. The multiple three tetratricopeptide repeat (TPR) domains of HOP mediate the simultaneous binding of Hsp90 and Hsp70.

**Table 1:** Co-chaperones of Hsp90.

Co-chaperone	Function
**Cdc37**	Interacts with protein kinases
**p23**	Facilitates the maturation of client proteins
**Aha1**	Stimulates Hsp90 ATPase activity
**SGT1**	Binds to Hsp90 *N*-terminal domain, and inhibits Hsp90 ATPase activity
**HOP**	Delivers steroid hormone receptor clients to Hsp90, and also mediates the binding of Hsp90 and Hsp70
**TAH1**	TPR containing protein, inhibits Hsp90 ATPase activity by forming cochaperone complex with PIH1
**CHIP**	Is an E3 ubiquitin ligase, and regulates the balance of folding/degradation for Hsp90 clients
**FKBP51/52**	Mediates the interaction of steroid receptor with Hsp90

TAH1, TPR-containing protein associated with Hsp90; TPR, TetratricoPeptide Repeat; PIH1, protein interacting with Hsp90; CHIP, Carboxyl terminus of Hsc70-interacting protein; FKBP, FK506-binding protein.

Post-translational modifications, such as phosphorylation, acetylation, and nitrosylation, also play critical roles in the regulation of Hsp90 function (Table 2). Phosphorylation of several serine, threonine and tyrosine residues is known to influence the function of Hsp90. For example, phosphorylation of Hsp90 at Ser 226/255 prevents the binding of Hsp90 to apoptotic peptidase activating factor 1 (Apaf-1), which thereby induces cytochrome c-induced apoptosome assembly [6]. Conversely, the phosphorylation is absent in leukemia cells constitutively expressing tyrosine kinases, such as BCR-ABL and Flt3/D835Y. This leads to Hsp90-Apaf1 association and subsequently abrogates cytochrome c-induced apoptosome assembly. Hsp90 acetylation is also found at many sites [19,20]. Mutation of Hsp90 at residue K294 displays reduced viability and chaperone function due to the deacetylation. Histone deacetylase (HDAC) inhibitors and knockdown of HDAC6 induce Hsp90 acetylation and inhibit its activity [21,22]. Nitrosylation is another post-translational modification of Hsp90 function; for instance, nitrosylation of Hsp90 at Cys597 inhibits chaperone activity in endothelial cells [23]. Collectively, this knowledge of the diverse regulation of Hsp90 function not only provides insight on Hsp90 chaperone machinery, but also has potential therapeutic implications.

**Table 2:** Post-translational modification of HSP90.

Post-translational modification	Function
**Phosphorylation**	Hsp90 has been identified as a substrate of prtoein kinases such as BRAF, CK2, Src, PP5, WEE1. The phosphorylation status of Hsp90 affects its function
**Acetylation**	About 11 lysine residues in Hsp90 have been found to be acetylated
**Nitrosylation**	Nitrosylation of Cys597 inhibits the ATPase activity of Hsp90

## 3. Hsp90 in Hematologic Malignancies

**Figure 1:**
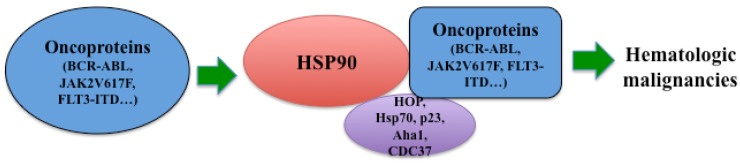
Role of Hsp90 in hematological malignancies. Hsp90 stabilizes oncogenic kinases including BCR-ABL, JAK2V617F and FLT3-ITD, *etc*. Co-chaperones, such as HOP, Hsp70, p23, Aha1 and CDC37, assist Hsp90 for its chaperone activity. Thus, Hsp90 is crucial for the development of many hematologic malignancies.

HSP90 is a ubiquitously expressed protein chaperone required for the stabilization of multiple oncogenic kinases, such as BCR-ABL [4], FLT3 [24] and JAK2 [5,25]. Therefore, Hsp90 plays a critical role in the development of leukemia by regulating survival and proliferation of leukemia cells (Figure 1).

### 3.1. Hsp90 and Philadelphia Chromosome-Positive Leukemia

The human Philadelphia chromosome (Ph^+^) arises from a reciprocal translocation between chromosomes 9 and 22 [(9;22)(q34;q11)], thereby resulting in the formation of the BCR-ABL oncogene that encodes a constitutively activated tyrosine kinase [26]. The most well known BCR-ABL tyrosine kinase inhibitor (TKI), imatinib mesylate, induces a complete hematologic and cytogenetic response in the majority of chronic myeloid leukemia (CML) patients in chronic phase [27,28,29]. However, resistance to TKI has been frequently observed, especially in patients with advanced-stage disease [30]. One critical reason is the occurrence of point mutations in the kinase domain of BCR-ABL, especially a well-known T315I mutation, resulting from a threonine to isoleucine substitution at position 315 [30]. A recent study showed that BCR-ABL forms a complex with Hsp90 and its co-chaperone p23 in K562 chronic myeloid leukemia cells [3]. Hsp90 inhibitor geldanamycin (GA) decreases the association of BCR-ABL with Hsp90 and p23 and increases its association with the chaperones Hsp70 and HOP. This leads to BCR-ABL degradation mediated by the proteasome, based on the fact that this degradation is blocked with addition of proteasome inhibitors [3]. Therefore, this result provides an alternative strategy for overcoming resistance caused by mutations in the BCR-ABL kinase domain. To determine whether inhibition of Hsp90 could induce degradation of TKI-resistant BCR-ABL, hematopoietic cells expressing BCR-ABL-T315I or E255K (resulting from a glutamic acid to lysine substitution at position 255) were treated with Hsp90 inhibitor GA and 17-allylaminogeldanamycin (17-AAG). Both compounds induced the degradation of BCR-ABL mutants and inhibited cell growth, with a trend indicating more potent activity against mutant BCR-ABL proteins [31]. Our previous data also confirmed that inhibition of Hsp90 caused degradation of wild type and mutant BCR-ABL proteins [4]. We found that IPI-504, a hydroquinone hydrochloride derivative of 17-AAG, efficiently induced dissociation of BCR-ABL and Hsp90 in BCR-ABL-expressing 32D cells as quickly as 30 min after the treatment [4]. In wild type BCR-ABL or BCR-ABL-T315I mutant induced CML mice [32], we also observed that treatment with IPI-504 alone significantly prolonged the survival of mice with wild-type BCR-ABL-induced CML, and this therapeutic effect was much more apparent in the survival of mice with BCR-ABL-T315I-induced CML [4]. The markedly prolonged survival of the IPI-504-treated BCR-ABL-T315I-induced CML mice correlates with the more pronounced *in vivo* degradation of the mutant BCR-ABL than that of wild type BCR-ABL. That is because the stability of BCR-ABL is shown *in vitro* to be more dependent on Hsp90 when it carries imatinib-resistant mutations [31]. Using our BCR-ABL induced B-acute lymphoid leukemia (B-ALL) mouse model, we also examined the effect of Hsp90 inhibitor on B-ALL, as it does not respond well to BCR-ABL kinase inhibitors. As expected, a similar effect was observed in CML. IPI-504 treatment dramatically delayed the development of B-ALL induced by BCR-ABL-T315I mutant. Interestingly, we found that although IPI-504 was active in B-ALL, it had a much stronger effect on CML mice [4]. The observation that Hsp90 was more strongly induced in myeloid cells than in lymphoid cells might provide the molecular basis for these different effects of Hsp90 inhibition on CML versus B-ALL. However, the detailed mechanisms need to be further investigated.

It is widely accepted that targeting CML stem cells is essential for curing CML, because CML stem cells survive and persist under TKI treatment and are responsible for disease relapse. Like normal hematopoietic stem cells (HSCs), LSCs can be defined as a specific cell population that can self-renew and has the ability to initiate cancer development [33,34,35]. Bonnet and Dick first identified and characterized LSCs from human AML samples [36]. They isolated CD34^+^CD38^−^ cells and transplanted them into non-obese diabetic mice with severe combined immunodeficiency disease (NOD/SCID) mice. They found that these cells not only initiate AML development in NOD/SCID mice but also differentiate *in vivo* into leukemic blasts [36]. More importantly, serial transplantation demonstrated that these cells have a capacity to self-renew and transfer AML disease into secondary recipients. Therefore, this study showed for the first time that LSCs in these AML patients were characterized by an ability to self-renew and recapitulate the disease. These LSCs also exhibited CD34^+^CD38^−^ phenotype, which are the same cell-surface markers as those on normal human primitive cells. In CML mice, HSCs harboring BCR-ABL function as LSCs, as sorted BCR-ABL-expressing Lin^-^Sca-1^+^c-Kit^+^ cells transferred CML into secondary recipients [37,38], but not other CML cell populations expressing differentiation markers [38]. Using this mouse CML stem cell model, bone marrow cells from mice with T315I-induced CML were cultured under the conditions that support survival and growth of stem cells and treated with IPI-504. We found that compared with the untreated group, IPI-504 treatment had a dramatic inhibitory effect on LSCs [4], indicating Hsp90 inhibition could efficiently eliminate LSCs. Our recently published result has indicated that hypoxia inducible factor 1α (HIF1α) plays a crucial role in survival and maintenance of LSCs [39]. Deletion of HIF1α impairs the propagation of CML through impairing cell cycle progression and inducing apoptosis of LSCs. Compared to normal HSCs, LSCs appear to be more dependent on the HIF1α pathway [39]. Interestingly, Hsp90 is critical for stabilizing HIF1α. Inhibition of Hsp90 by 17-AAG impaired HIF1α stability in a von Hippel-Lindau (VHL) independent manner, and blocked cancer cell invasiveness [40]. The next generation small molecule Hsp90 inhibitor EC154 can target hypoxia inducible factor [41]. These studies imply that HIF1α might be another mediator of Hsp90 function in LSCs. Together; these studies demonstrate that inhibition of Hsp90 can effectively inhibit the survival and proliferation of LSCs and provide a therapeutic strategy for eradicating LSCs in CML.

### 3.2. Hsp90 and Philadelphia Chromosome-Negative Myeloproliferative Neoplasms

Like CML, other myeloproliferative neoplasms (MPNs), such as polycythaemia vera (PV), essential thrombocythaemia (ET) and primary myelofibrosis (PMF), are also clonal disorders of multipotent hematopoietic progenitors [42]. The identification of the JAK2V617F mutation uncovered the genetic cause for these diseases [43,44,45,46], thereby leading the field of Philadelphia-negative MPNs into the era of targeted therapy. JAK2 is a cytoplasmic non-receptor tyrosine kinase. The JAK2V617F mutation results in a single amino acid substitution: valine to phenylalanine. As valine 617 is critical for JAK2 autoinhibition, this substitution disrupts autoinhibition and results in constitutive kinase activity [42], which activates multiple downstream signaling pathways including signal transducer and activator of transcription (STAT), mitogen activated protein kinase (MAPK) and phosphatidylinositol 3-kinases (PI3K)-AKT pathways. Currently, several JAK2 inhibitors are being tested in clinical trials for patients with MPNs. These drugs act by blocking the proliferation of neoplastic cells through blocking the JAK2 signaling pathways. Continuous treatment with the JAK1/2 inhibitor, ruxolitinib, was associated with marked and durable reductions in splenomegaly and disease-related symptoms of PMF patients, and about 28% of patients in the ruxolitinib group got at least 35% reduction in spleen size at week 48 [47]. Ruxolitinib has become the first FDA approved drug for the treatment of patients with intermediate and high-risk myelofibrosis, and has limited efficacy of JAK2 inhibitors in a large amount of MPN patients. In addition, the occurrence of drug resistance mutations occurs as a result of the treatment with ATP-mimetic inhibitors. Several JAK2 mutations within the kinase domain have been identified [5]. Therefore, alternative therapeutic approaches for MPDs are necessary. Targeting JAK2 protein stability is one of these strategies.

Recently JAK2 has been shown to bind to Hsp90 [25]. JAK2V617F expressing mouse Ba/F3 and human UKE-1 leukemia cells displayed higher sensitivity to a non-anasamycin Hsp90 inhibitor PU-H71. PU-H71 treatment was associated with induction of apoptosis and inhibition of proliferation by abrogating phosphorylation of JAK2 and its related downstream pathways, including STAT3/5 and MAPK signaling. Additionally, PU-H71 caused dose-dependent degradation of JAK2 *in vitro* and *in vivo*. PU-H71 also improved survival of mice with MPLW515L-induced MPN, suggesting Hsp90 inhibitors might have a broader therapeutic window than JAK2 inhibitors. Importantly, Hsp90 inhibition may overcome the resistance resulting from JAK2 mutants, as Hsp90 inhibitors promoted the degradation of both wild type and mutant JAK2 [5]. Thus, Hsp90 is a promising therapeutic target in JAK2-driven MPNs.

### 3.3. Hsp90 and Acute Myeloid Leukemia

Although great improvements have been achieved in acute myeloid leukemia (AML) treatment over the last several decades, the majority of patients still die of this disease [48]. Advances in understanding of the molecular mechanisms of this disease provide a promise for molecular therapeutics. A number of molecular markers, such as FMS-like tyrosine kinase 3 (FLT3) and Janus kinase (JAK-2), have been identified [49]. FLT3 is a receptor tyrosine kinase expressing in early hematopoietic progenitor cells and plays a key role in hematopoietic development [50,51]. Upon stimulation with FLT3 ligand, FLT3 dimerises and undergoes autophosphorylation; thereby upregulating its tyrosine kinase activity and activating multiple downstream pathways, such as PI3K/Akt and Ras signaling [52,53]. FLT3 is expressed at high levels in 70–100% of cases of AML and virtually all cases of B-ALL [54]. Mutations of FLT3 are observed in approximately 33% patients with AML and are associated with adverse clinical outcomes [55]. These mutations fall into two main classes: FLT internal tandem duplication (FLT-ITD) and FLT tyrosine kinase domain (FLT-TKD). Therefore, targeting the kinase activity of FLT3 with inhibitors is an attractive therapeutic option. This will help in the development of several FLT3 inhibitors that compete for ATP binding in the ATP-binding pocket of the kinase domain of FLT3. Similar to imatinib for BCR-ABL, these preclinical and clinical inhibitors bind to the active or inactive FLT3, and are greatly influenced by changes in the binding pocket of FLT3.

Hsp90 is highly expressed in AML cells [56], and it is the main chaperone required for the stabilization of multiple oncogenic kinases involved in the development of AML [57]. FLT3 appears to require Hsp90 for proper folding and stability [6]. Interestingly, a recent study characterized the expression profiles of various HSPs in AML from 75 consecutive patients, and found that although HSP levels in primary human AML cells vary significantly between patients, FLT3-ITD is strongly associated with a higher expression level of HSPs [58]. Therefore, Hsp90 inhibitors have profound inhibitory effects on AML cells with FLT3-ITD [58]. This feature makes it possible to specifically target human leukemia with mutated FLT3 kinase by Hsp90 inhibitors. Herbimycin A (HA) is an ansamycin derivative and inhibits the constitutive tyrosine phosphorylation of FLT3-ITD but not wild type FLT3 [59]. Immunoprecipitation analysis showed that FLT3-ITD but not wild type FLT3 formed a complex with Hsp90, and inhibition of Hsp90 by HA causes the dissociation of FLT3-ITD and Hsp90. Similarly, treatment of human AML MV4-11 cells with another Hsp90 inhibitor 17-AAG attenuated the levels of FLT3-ITD by inhibiting its association with Hsp90 and inducing the poly-ubiquitylation and degradation of FLT3-ITD [24]. The combination of 17-AAG and FLT3 kinase inhibitor PKC412 caused synergistic effects and induced apoptosis in MV4-11 cells and primary AML blasts. A recent study shows that the E3 ubiquitin ligases c-Cbl and Cbl-b facilitated the polyubiquitination of autophosphorylated FLT3-ITD, thereby causing degradation of FLT3-ITD and significantly enhancing the 17-AAG-induced decline in autophosphorylated FLT3-ITD [60]. Conversely, overexpression of loss-of-function mutants of both c-Cbl (c-Cbl-R420Q) and Cbl-b (Cbl-b-C373A) together in 32D cells conferred these cells the resistance to cytotoxicity of 17-AAG by inhibiting the 17-AAG-induced degradation of FLT3-ITD. These results suggest that c-Cbl and Cbl-b play an important role in Hsp90 inhibitor-induced degradation of FLT3-ITD in leukemic cells. Furthermore, published results indicate that HSPs are released to the extracellular compartment, and play an important role in anti-tumor responses [61,62]. A recent study also shows that AML patients have an increased level of serum Hsp90/70, and a lower level of HSPs in serum are associated with a prolonged survival during treatment with all-trans retinoic acid (ATRA) plus valproic acid and theophyllin [63]. These results suggest that the serum HSPs might be a prognostic indicator in AML treatment.

### 3.4. Hsp90 and Other Blood Cancers

Higher Hsp90 expression is also observed in other blood cancers, including multiple myeloma (MM) and most of non-hodgkin’s lymphoma [64,65], suggesting that Hsp90 inhibition provides a potential strategy for treating these diseases. Multiple myeloma is an incurable malignancy of plasma cells, and is characterized by an abnormal clonal plasma cell infiltration in the bone marrow, as the bone marrow microenvironment is essential for maintaining plasma cell survival [66]. The molecular pathogenesis of MM is poorly understood. Nearly half cases have chromosome translocations that juxtapose genes including *CCND1*, *CCND3*, *MAF*, *MAFB*, *FGFR3* and *MMSET* with the immunoglobulin heavy chain locus (*IgH*), causing overexpression of these genes [67]. However, malignant progression of MM requires activation of *MYC, KRAS, NRAS* and the nuclear factor-kB pathway (NF-kB) [67,68]. Using 38 MM patients and performing whole-genome sequencing and whole-exome sequencing, a recent study further revealed the genetic complexity of MM [69]. Importantly, a serial of studies indicate that the components of these deregulated pathways, such as CCND1 [70], RAS [71], and NF-kB [72], are the Hsp90 client proteins. Therefore, targeting these deregulated pathways by inhibiting Hsp90 might contribute significantly to the treatment of MM. Moreover, both knockdown of Hsp90 by shRNA and inhibition of Hsp90 by its inhibitors alter the level of STAT3 and phospho-Erk and induce the apoptosis of multiple myeloma cells [73]. Furthermore, mantle cell lymphoma (MCL) is a distinct subtype of non-Hodgkin lymphoma, arising from cells that surround the germinal center of the B-cell follicle. MCL shows aberrant expression of *CCND1* gene. Previous study has shown that Hsp90 inhibition induces cell cycle arrest and cell death in MCL cells, and these effects are associated with the downregulation of CCND1 [70]. On the other hand, diffuse large B-cell lymphoma (DLBCL) is a major subtype of B-cell lymphoma, and is characterized by large B cells [74]. Multiple genetic alterations including chromosome translocations (BCL6, MYC) and mutations of tumor suppressor genes (P53) have been identified in DLBCL [74]. Hsp90 interacts with BCL6 in DLBCL cells and stabilizes BCL6 mRNA and protein. Hsp90 inhibitors selectively kill diffuse large B cell lymphomas (DLBCLs) that depend on the BCL-6 transcriptional repressor [75]. Finally, B-chronic lymphocytic leukemia (B-CLL) is characterized by the accumulation of small B cells that express the CD5 antigen, and lacks a common genetic aberration that is amenable to be targeted theraupeutically [76]. A recent study of normal B cell versus CLL shows differences in B-cell receptor (BCR) signaling, suggesting a selective therapeutic strategy for B-CLL [77]. Src family kinase Lyn, a critical molecule of BCR pathway, is overexpressed in B-CLL cells and displays a high constitutive activity. Hsp90 facilitates this aberrant activity through tight binding to Lyn. Hsp90 inhibition by GA causes disruption of Hsp90/Lyn complex, resulting in the inactivation of Lyn in B-CLL cells [78].

Together, given the important role of Hsp90 in regulating the critical molecules that are essential for various hematologic malignancies, Hsp90 has become a common therapeutic target for these cancers (Table 3).

**Table 3:** HSP90 clients in hematologic malignancies.

Hematologic malignancies	Hsp90 clients
**CML, B-ALL**	BCR-ABL
**MPN**	JAK2V617F
**AML**	FLT3-ITD
**Multiple myeloma **	CCND1, RAS, MYC, NF-kB pathway, STAT3
**B-chronic lymphocytic leukemia**	Lyn, BCR pathway
**Mantle cell lymphoma**	CCND1
**Diffuse large B-cell lymphoma**	BCL6, BCL2, MYC, P53

## 4. Chemical Inhibitors of Hsp90

**Figure 2:**
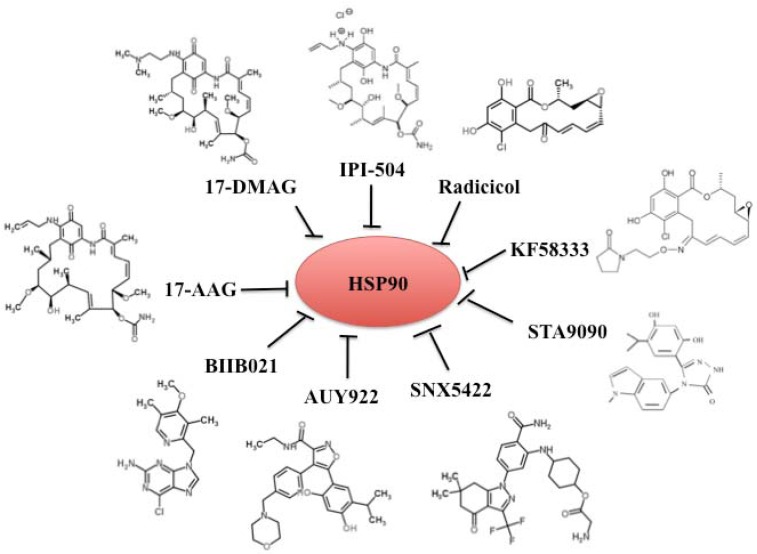
Hsp90 inhibitors. Hsp90 inhibitors are classified into three groups: benzoquinone ansamycins 17-AAG and its derivatives 17-DMAG and IPI-504, radicicol and its derivates, and small synthetic inhibitors.

The studies described above highlight the promise of Hsp90 as a novel therapeutic target in cancer treatment. Basic biology of Hsp90 provides useful information for translational drug development which has resulted in development of several different Hsp90 inhibitors. These inhibitors can be classified into three groups: (1) benzoquinone ansamycins 17-AAG and its derivatives 17-DMAG and IPI-504, (2) radicicol and its derivates, and (3) and small synthetic inhibitors. By mimicking the ATP binding structure in the N-terminal domain of Hsp90, these inhibitors selectively block the ATP binding and hydrolysis thus inhibiting the chaperone functions. This subsequently interferes with the protein stability and folding of Hsp90 client proteins, resulting in antitumor activity. Currently, 17 Hsp90 inhibitors are being tested in clinical trials (Table 4). Here, we focused on several classical inhibitors in each group (Figure 2).

**Table 4:** HSP90 inhibitors.

Inhibitors	Properties	Group	Clinical trial phase
17-AAG (tanespimycin)	Well tolerated; limited oral bioavailability and solubility	Benzoquinone ansamycin	II/III
17-DMAG (alvespimycin)	Well tolerated; soluble	Benzoquinone ansamycin	I
IPI-504 (retaspimycin)	Highly soluble and well tolerated	Benzoquinone ansamycin	III
IPI493	Primary active, long-lived metabolite of 17-AAG; low solubility	Benzoquinone ansamycin	I
Radicicol	Macrocycli antibotic; poorly soluble and unstable	Radicicol	None
KF58333	Highly soluble and stable	Radicicol	None
BIIB021 (CNF2024)	An oral purine scaffold compound	Small molecular inhibitor	II
AUY922	An isoxazole resorcinol derivative	Small molecular inhibitor	II
STA-9090	A resorcinol-containing triazole compound; highly soluble	Small molecular inhibitor	II
SNX-5422/SNX-2112	A pyrazole-containing compound; highly soluble	Small molecular inhibitor	I
KW-2478	A resorcinol analog; highly soluble	Small molecular inhibitor	I/II
AT13387	A resorcinol-containing compound	Small molecular inhibitor	I
XL888	Highly soluble	Small molecular inhibitor	I
NVP-HSP990	An isoxazole resorcinol derivative	Small molecular inhibitor	I
MPC-3100	An oral purine scaffold compound	Small molecular inhibitor	I
ABI-010	Developed using nanoparticle albumin-bound (nab) technology	Small molecular inhibitor	I

### 4.1. Benzoquinone Ansamycins 17-AAG and Its Derivatives 17-DMAG and IPI-504

17-AAG (also called tanespimycin) is an analogue of GA. It can induce cytosolic accumulation of cytochrome c and cleavage of caspase-9 and caspase-3, thereby triggering apoptosis of leukemia cells [79]. In addition, 17-AAG down-regulates intracellular BCR-ABL and Raf oncogene serine/threorine (c-Raf) proteins and reduces activity of Akt kinase in K562 cells [79]. It is the first-in-class Hsp90 inhibitor tested in Phase I clinical trials. 17-AAG monotherapy was well tolerated and demonstrated activity across all doses tested in heavily pretreated patients with relapsed/refractory multiple myeloma [80]. Although the phase I clinical trial showed encouraging initial results, 17-AAG displayed limited oral bioavailability and solubility. These drawbacks might block the efficacy of 17-AAG in many cancers, as observed in many phase II clinical trials. The effect of 17-AAG on RAF kinase expression was short-lived, and no objective anti-melanoma response was observed [81]. 17-AAG also did not achieve objective response in metastatic, hormone-refractory prostate cancer, and clear cell or papillary renal cell carcinoma patients [82,83]. Moreover, at clinically tolerable doses 17-AAG has little effect on relapsed leukemia and exhibits limited activity in combination with cytarabine [84].

The failure of these trials highlights the the need for more potent Hsp90 inhibitors. New analogues of 17-AAG, 17-DMAG and IPI-504, have been developed [31]. 17-DMAG (also called alvespimycin) came from screening assays for the potent geldanaymycin analog, and displayed inhibitory function on HL60-TB leukemia and other cancer cells, which were relatively insensitive to 17-AAG [85]. 17-DMAG is active when taken orally, and combination of 17-DMAG and the mitogen-activated protein kinase/ERK kinase 1/2 inhibitor potently induces apoptosis of imatinib resistant BCR-ABL expressing leukemia cells by sustained extracellular signal regulated kinase (ERK1/2) inactivation and B-cell lymphoma extra large (Bcl-xL) down-regulation [86]. 17-DMAG functions as a nuclear factor kappa-light-chain enhancer (NF-κB) inhibitor, and is cytotoxic to CLL but not to normal lymphocytes [87]. Treatment with 17-DMAG led to depletion of the Hsp90 client protein IκB kinase (IKK) and decreased NF-κB target gene transcription. Further, 17-DMAG significantly prolonged the survival in a transplant mouse model. 17-DMAG also showed a potential therapeutic effect in Non-small-cell lung cancer (NSCLC) patients with epidermal growth factor receptor (EGFR) mutations with or without EGFR-tyrosine kinase inhibitors (EGFR-TKIs) resistance [88]. Western blot analysis revealed that 17-DMAG treatment inhibited the activation of EGFR, Akt, and MAPK pathways in EGFR-mutant cell lines but not in EGFR-wild type cell lines. Cleaved PARP expression confirmed apoptosis in response to 17-DMAG treatment in EGFR-mutant cell lines but not in EGFR-wild type cell lines. Recent clinical trial using 24 patients with advanced AML showed that 17-DMAG was well tolerated, and pharmacodynamic analyses revealed increased apoptosis and Hsp70 levels when compared with baseline within marrow blasts [89]. The effect of 17-DMAG is of great interest clinically, but further clinical trials are required to validate its efficacy.

IPI-504 (also called retaspimycin) is highly soluble in water and generally well tolerated. We have showed that IPI-504 efficiently inhibited the development of CML induced by wild type BCR-ABL and mutant BCR-ABL-T315I [4]. Recent studies have demonstrated that IPI-504 has high activity in diffuse large B-cell lymphoma, and mantle cell lymphoma [90,91]. Given the facts that IPI-504 has shown activity in NSCLC and gastrointestinal stromal tumor in phase I/II trials [92], future trials are of clinical interest to evaluate the dosing schedules and activity of IPI-504 in blood cancers.

### 4.2. Radicicol and Its Derivates

Radicicol is a macrocyclic antibiotic produced by fungi, and is originally described as tyrosine kinase inhibitor because of its ability to inhibit the signal transduction of oncogene products, such as K-ras and Src [93]. A recent work has produced improved radicicol and its analogues that show promising Hsp90 inhibitory activity *in vitro * [94]. Like ansamycins**, **radicicol can inhibit the ATPase activity by binding to the N-terminal ATP pocket and deplete the Hsp90 client signaling molecules in cells. However, radicicol is poorly soluble and unstable in animals and shows little or no activity in animals. The oxime derivatives of radicicol showed potent antitumor activities against human tumor xenograft models [93]. Treatment with radicicol oxime derivatives results in binding to Hsp90 and destabilizing of Hsp90 client proteins in the tumor. Oxime derivatives of radicicol, KF58333, induced the expression of glycophorin A in K562 cells, and resulted in an accumulation of cells in G1 phase by down-regulating the level of cell cycle-dependent kinases 4 and 6 (CDK4/6) and up-regulating cell cycle-dependent kinase inhibitor p27 (Kip1) protein [94]. KF58333 treatment also depleted BCR-ABL, Raf-1, and cellular tyrosine phosphorylated proteins in K562 cells by affecting Hsp90-p23-BCR-ABL complex. Importantly, administration of KF58333 prolonged the survival time of SCID mice inoculated with K562 cells [94]. These results suggest that radicicol and its derivatives might have therapeutic potential for cancer treatment. However, these have not entered clinical development, possibly due to their severe side effects [95].

### 4.3. Synthetic Small-Molecular Inhibitors

Crystal structure of Hsp90 has helped in designing synthetic small-molecular inhibitors. BIIB021/CNF2024 is the first synthetic inhibitor of Hsp90 tested in a clinical trial. It is an oral purine scaffold compound and binds competitively with geldanamycin in the ATP-binding pocket of Hsp90 [96]. A recent study demonstrated that BIIB021 selectively induced cell death in Hodgkin’s lymphoma cells but not in lymphocytes from healthy individuals at low nanomolar concentrations in combination with doxorubicin and gemcitabine. In this process, NF-κB might mediate the function of BIIB021. Furthermore, HSP90 inhibition sensitizes Hodgkin’s lymphoma cells for natural killer (NK) cell-mediated killing via up-regulation of ligands involved in activating NK cell receptors [97]. BIIB021 also displayed higher activity in several human tumor xenograft models by inducing the degradation of Hsp90 client proteins and up-regulated expression of the heat shock proteins Hsp70 and Hsp27 [98]. In several completed phase I trials, BIIB021 appeared to be well tolerated [99]. A phase II evaluation of BIIB021 in hormone receptor positive metastatic breast cancer is ongoing.

A second class of synthetic Hsp90 inhibitors is the isoxazole resorcinol derivative AUY922. AUY922 treatment causes downregulation of multiple survival pathways and strong upregulation of Hsp70 in a large set of primary multiple myeloma tumor samples and in MM cell lines [65]. There are currently several ongoing phase I/II trials for various types of cancers.

SNX-2112 and SNX-5422 belong to small molecule class based on the 6,7-dihydro-indazol-4-one scaffold. These inhibitors showed more potent activity than 17-AAG orally. SNX-2112 inhibited proliferation induced by interleukin-6 and insulin-like growth factor-1, and induced apoptosis via caspase-8, 9, 3 and poly (ADP-ribose) polymerase cleavage. Additionally, SNX-2112 affected bone marrow microenvironment to block angiogenesis and osteoclastogenesis [100].

In a phase I clinical trial involving 33 patients with refractory solid tumor malignancies and lymphomas, SNX-5422 was well tolerated by all patients with refractory solid tumor malignancies and lymphomas when administered orally twice a week. Pharmacodynamic studies showed inhibition of Hsp90 [101]. However, SNX-5422 causes ocular toxicity in animal models and in phase I clinical trials, and the development of this drug has been discontinued.

STA-9090 (also called ganetespib) is a resorcinol-containing triazole compound. STA-9090 exhibited potent *in vitro* and *in vivo* activity in a range of solid and hematological tumor cells that are dependent on JAK2 activity for growth and survival. Treatment with STA-9090 resulted in a sustained depletion of JAK2, including the constitutively active JAK2 (V617F) mutant, with subsequent loss of STAT activity and reduced STAT-target gene expression [102]. STA-9090 also had broad range activity against mast cells expressing WT or mutant Kit [103]. The results from a Phase I trial showed that STA-9090 was well tolerated in over 500 patients, and without evidence of severe liver or common ocular toxicities typically seen after administration of other Hsp90 inhibitors. The most common adverse event associated with STA-9090 has been transient mild or moderate diarrhea, which is manageable with standard supportive care.

## 5. Conclusions

Preclinical and clinical studies during this past decade have uncovered the central role of Hsp90 in diverse cellular processes and provided encouraging results for Hsp90 inhibitors in myeloid leukemia, multiple myeloma, lymphoma and other solid cancers, such as castrate refractory prostate cancer and non-small cell lung carcinoma. However, there are a series of issues that need to be addressed.

Cancer drug therapy is often compromised by the development of resistance. Acquired resistance to Hsp90 inhibitors has been observed in glioblastoma cells. Four 17-AAG-resistant glioblastoma cell lines were obtained after continuous exposure to increased 17-AAG concentrations. Cross-resistance was found with other benzoquinone ansamycins but not with the structurally unrelated HSP90 inhibitors, such as radicicol, BIIB021 and NVP-AUY922. This resistance might correlate with low endogenous expression of NAD(P)H/quinone oxidoreductase I (NQO1), which is critical for the Hsp90 binding ability of 17-AAG [104,105]. Another underlying mechanism is the inactive *NQO1* polymorphism [104]. Furthermore, given the fact that all of the current inhibitors recognize the ATP binding structure in the N-terminal domain of Hsp90, mutations in this region with no effect on Hsp90 function might confer resistance to Hsp90 inhibitors by blocking their binding to Hsp90. A single mutation in which a leucine in the N-terminal domain of Hsp90 from the fungus was replaced by isoleucine caused lower affinity for radicicol and generated resistance to radicicol [106]. Therefore, we require alternative strategies for targeting Hsp90 to overcome drug resistance. One of these strategies is to identify inhibitors that recognize the middle domain or C-terminal domain of Hsp90. Further structural analysis of Hsp90, which is hampered by the high intrinsic flexibility of structural conformation of Hsp90, will facilitate this method. Additionally, inhibiting Hsp90/co-chaperone interactions might be an alternative approach. A recent study demonstrated that natural product celastrol, disrupted Hsp90-Cdc37 interaction by blocking the critical interaction of Glu33 (Hsp90) and Arg167 (Cdc37) in the superchaperone complex. Importantly, although celastrol did not interfere with ATP binding to Hsp90, it induced apoptosis *in vitro* and significantly inhibited tumor growth. This study has proved the possibility of targeting the interaction of Hsp90 and its co-chaperones such as CDC37, Aha1 and p23 as a way to treat cancers. Higher expression of these co-chaperones has been obtained in cancer cells, and their inhibition has been shown to increase the efficacy of Hsp90 inhibitors [2,107].

**Figure 3:**
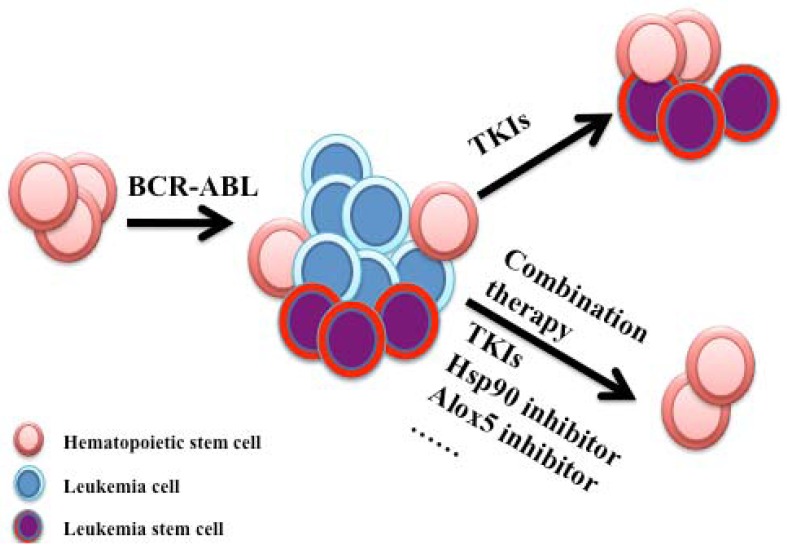
Strategies for targeting leukemia stem cells. BCR-ABL transforms a hematopoietic stem cell into a leukemia stem cell, subsequently giving rise to CML. Treatment with TKIs inhibits BCR-ABL activity and eliminate proliferative leukemia cells. However, residual leukemia stem cells are persistent with TKI treatment. Combination therapy with a TKI and a Hsp90 inhibitor or Alox5 inhibitor will be a promising therapeutic strategy for targeting leukemia stem cells.

Targeted molecular therapies have become more and more important for the majority of hematologic malignancies. Eradicating the leukemic stem cell population is an attractive therapeutic strategy for curing these diseases. Although our studies involving CML stem cells and Hsp90 highlight the essential role of Hsp90 in cancer stem cells [4], the role of Hsp90 still remains unknown in cancer stem cells of other hematologic malignancies. Moreover, given the fact that resistance to conventional therapy is very common in current therapies for hematologic malignancies and that Hsp90 has been implicated in the resistance of leukemia cells to potential therapeutic agents [4], combination of Hsp90 inhibition with conventional therapy provides a promising and effective therapeutic strategy for cancer treatment (Figure 3). Further clinical studies are needed to clarify the efficacy and toxicity of Hsp90 inhibitors in the treatment of human hematologic malignancies, where Hsp90 inhibitors should be combined with conventional chemotherapy. A comprehensive understanding of Hsp90 structure and function will drive the development of Hsp90 inhibitors, which will eventually contribute to effective cancer therapy in the future.
